# Integrated Multi-Omics Characterization of the Salt-Sensitive Mutant *sss1* in Soybean

**DOI:** 10.3390/plants15111695

**Published:** 2026-05-30

**Authors:** Ziyun Chen, Xi Li, Jinyang Zhang, Chunyu Liu, Liyan Wan, Guangcheng Bai, Peiying Hao, Ping Deng, Fengjie Yuan

**Affiliations:** 1 College of Life Sciences, China Jiliang University, Hangzhou 310018, China; 2 Zhejiang Academy of Agricultural Sciences, Hangzhou 310021, China; 3 Xianghu Laboratory, Hangzhou 311231, China; 4 College of Life Sciences, Nankai University, Tianjin 300071, China

**Keywords:** *Glycine max*, salt stress, ion homeostasis, metabolomics, transcriptomics, phytohormones

## Abstract

Soil salinity is a major constraint on soybean (*Glycine max*) production. While ionic toxicity is a primary factor, the integration of metabolic and hormonal responses remains unclear. Here, we characterize the salt-sensitive mutant, *soybean salt-sensitive 1* (*sss1*), using physiological, metabolomic, and transcriptomic analyses. We demonstrate that the primary defect in *sss1* is disrupted ion homeostasis, characterized by excessive Na^+^ accumulation, impaired K^+^ retention, and a high Na^+^/K^+^ ratio. Multi-omics integration revealed that *sss1* exhibits extensive metabolic reprogramming. Notably, mannose was identified as a potential hub linking carbohydrate metabolism and glycosylation; its reduction in the mutant suggests a metabolic vulnerability. Furthermore, the mutant showed a dual impairment of abscisic acid (ABA) and jasmonic acid (JA) signaling, evidenced by reduced hormone levels and downregulation of biosynthetic genes. Collectively, our results associate the *sss1* locus with systemic disruptions in ion homeostasis, hormone signaling, and metabolic reprogramming. This multi-omics landscape provides a foundation for the future cloning of *SSS1* and elucidating the molecular mechanisms underlying salt sensitivity in soybean.

## 1. Introduction

Soil salinity represents one of the most severe abiotic stresses confronting global agriculture, currently affecting approximately 20% of irrigated agricultural land and 33% of arable land worldwide [1,2]. This escalating environmental challenge poses a significant threat to crop yield and global food security, particularly as climate change exacerbates soil degradation [3]. Soybean (*Glycine max* L. Merr.), a globally vital legume, is a primary source of plant protein and vegetable oil, substantially contributing to the world’s nutritional supply [4]. However, soybean is classified as a glycophyte and is notably sensitive to salt stress, especially during the critical seedling and reproductive stages, which often results in substantial yield losses [5,6].

Salinity imposes a dual challenge on plants: initial osmotic stress, which reduces water uptake, and subsequent ionic toxicity, primarily caused by the excessive accumulation of Na^+^ ions and the disruption of K^+^ homeostasis [7,8]. This ionic imbalance is widely recognized as a primary cause of salt-induced damage, as the maintenance of a low cytosolic Na^+^/K^+^ ratio is a key determinant of salt tolerance in glycophytic plants [1,9]. To counteract ionic toxicity, plants have evolved sophisticated mechanisms to regulate ion balance. These include the active exclusion of Na^+^ at the root surface, the sequestration of Na^+^ into the vacuole to protect cytosolic enzymes, and the controlled long-distance transport of Na^+^ mediated by specific transporter families [10]. The HKT (High-Affinity K^+^ Transporter) family, particularly HKT1;1, plays a pivotal role in retrieving Na^+^ from the xylem sap to limit its translocation to the shoot, thereby protecting photosynthetic tissues [11,12]. Concurrently, NHX (Na^+^/H^+^ Exchanger) antiporters facilitate the compartmentalization of Na^+^ into vacuoles using the proton gradient generated by V-ATPase and PPase [13,14]. Furthermore, the maintenance of K^+^ homeostasis through AKT and KUP/HAK/KT transporters is equally critical, as K^+^ is essential for enzyme activation and osmotic adjustment [15,16]. Despite these known mechanisms, the specific genetic regulation of these transporters in soybean under salinity remains an area of active research [17,18,19].

Beyond ion regulation, salt stress triggers extensive transcriptional and metabolic reprogramming. Plants activate a complex signal transduction cascade that leads to the accumulation of compatible solutes, such as proline, glycine betaine, and sugars, which function as osmoprotectants to maintain cellular turgor [20,21]. Additionally, membrane lipid remodeling occurs to maintain membrane fluidity and integrity [22]. In soybean, the accumulation of specific metabolites, such as isoflavonoids and flavonoids, has been linked to antioxidant defense mechanisms that scavenge reactive oxygen species (ROS) generated under stress [23,24]. However, how these diverse metabolic processes are transcriptionally coordinated into a harmonized network to confer salt tolerance in soybean is still poorly understood [25].

Phytohormones act as central integrators of environmental signals and developmental processes. Abscisic acid (ABA) is the primary hormone regulating plant responses to osmotic stress. Upon salt exposure, ABA levels surge rapidly, triggering stomatal closure to minimize water loss and activating stress-responsive gene expression through the PYR/PYL-PP2C-SnRK2 signaling cascade [26,27]. Jasmonic acid (JA), traditionally associated with defense against herbivores and necrotrophic pathogens, has emerged as a critical regulator of abiotic stress tolerance. JA modulates root growth, antioxidant enzyme activities, and membrane stability, often acting synergistically with ABA [28,29]. Importantly, the crosstalk between ABA and JA is not isolated; it is embedded within a complex hormonal network that includes ethylene (ET), brassinosteroids (BR), and auxin [30,31]. For instance, ethylene signaling often interacts antagonistically or synergistically with JA to fine-tune stress responses [32]. Although significant progress has been made in understanding individual hormone pathways, the integration of this hormonal crosstalk with metabolic and transcriptional networks in soybean salt adaptation remains largely uncharacterized [33].

Recent advances in high-throughput omics technologies offer unprecedented opportunities to dissect these complex regulatory networks. Integrated analysis of transcriptomics and metabolomics allows for the identification of coordinated gene–metabolite networks and the discovery of key regulatory hubs that might be missed by single-omics approaches [34]. This strategy has been successfully applied in model plants like *Arabidopsis* and crops such as rice, maize, and wheat to unravel salt tolerance mechanisms [35,36,37]. In this study, we identified a novel salt-sensitive soybean mutant, designated *sss1*, from an ethyl methanesulfonate (EMS)-mutagenized population of the cultivar Williams 82. Compared to the wild type, the *sss1* mutant exhibits severe growth inhibition and a complete failure to maintain ion homeostasis under saline conditions. By employing an integrated multi-omics approach, combining physiological assays, untargeted metabolomics, and transcriptomics, we reveal that the salt sensitivity of *sss1* arises from the disruption of potential nodes that coordinate Na^+^/K^+^ balance with metabolic reprogramming and altered ABA/JA accumulation. Our findings provide new insights into the multilayered regulatory networks governing salt tolerance in soybean.

## 2. Results

### 2.1. Phenotypic and Physiological Characterization of Salt Tolerance in the sss1 Mutant

**Figure 1:**
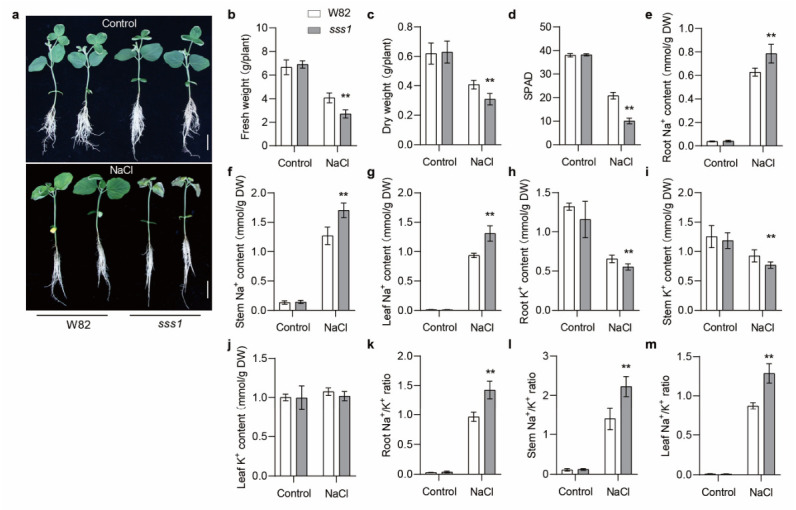
Phenotypic characterization, physiological parameters, and ion accumulation in W82 and *sss1* plants under salt stress. (**a**) Phenotypes of 2-week-old W82 and *sss1* seedlings grown on media without (Control) or with 150 mM NaCl (NaCl) for 5 days. Scale bar = 5 cm. (**b**,**c**) Fresh weight (**b**) and dry weight (**c**) of W82 and *sss1* plants under control and NaCl conditions. (**d**) SPAD values of W82 and *sss1* plants. (**e**–**j**) Na^+^ and K^+^ contents in roots (**e**,**h**), stems (**f**,**i**), and leaves (**g**,**j**) of W82 and *sss1* plants. (**k**–**m**) Na^+^/K^+^ ratios in roots (**k**), stems (**l**), and leaves (**m**). Data are presented as means ± SD (*n* = 8 in (**b**–**d**), *n* = 5 in (**e**–**m**)). Asterisks indicate statistically significant differences between W82 and *sss1* under the same condition (** *p* < 0.01, Student’s *t*-test).

The *sss1* mutant was isolated from EMS-mutagenized M_2_ populations of the soybean cultivar Williams 82 (W82). This mutant exhibited significantly reduced salt tolerance compared to the wild-type (WT) W82. While both genotypes displayed similar growth under control conditions, exposure to NaCl stress resulted in severe growth inhibition and leaf chlorosis in *sss1* plants, whereas W82 plants remained relatively healthy (Figure 1a). Consistent with these morphological observations, salt stress caused a significantly greater reduction in fresh weight, dry weight, and chlorophyll content (SPAD) in *sss1* than in W82 (Figure 1b–d).

To determine the physiological mechanism underlying the salt sensitivity of *sss1*, we analyzed ion accumulation and homeostasis. Under salt stress, the *sss1* mutant accumulated significantly higher concentrations of Na^+^ in roots, stems, and leaves compared to W82 (Figure 1e–g). Conversely, *sss1* plants exhibited a compromised ability to retain K^+^, with significantly lower K^+^ concentrations observed in roots and stems relative to W82, although no significant difference was found in leaves (Figure 1h–j). Consequently, the Na^+^/K^+^ ratios in the roots, stems, and leaves of *sss1* were significantly higher than those in W82 under salt stress conditions (Figure 1k–m). These results suggest that the salt sensitivity of *sss1* is likely caused by excessive Na^+^ accumulation and K^+^ loss, leading to ionic imbalance.

### 2.2. Metabolomic Profiling of Soybean Response to Salt Stress

**Figure 2:**
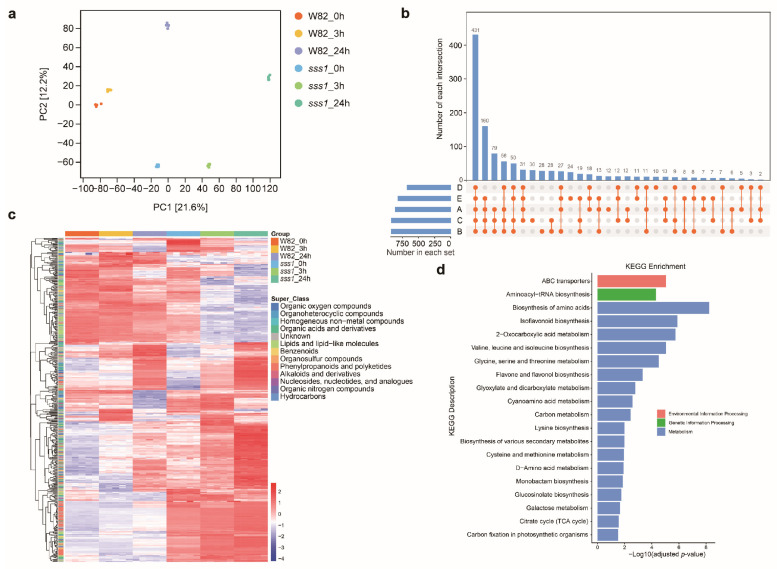
Metabolomic profiling and differential metabolite analysis between W82 and *sss1*. (**a**) Principal component analysis (PCA) score plot of all samples. PC1 and PC2 represent 21.6% and 12.2% of the total variance, respectively. (**b**) UpSet plot illustrating the overlap of differentially accumulated metabolites (DAMs) across five comparison groups. The specific comparisons are: (A) W82 vs. *sss1* at 0 h; (B) W82 vs. *sss1* at 3 h; (C) W82 vs. *sss1* at 24 h. Panels (D) and (E) represent the intra-group comparisons (replicates) for W82 at 3 h and 24 h, respectively. (**c**) Hierarchical clustering heatmap of the abundance of 431 DAMs. The color scale represents the relative abundance (z-score). (**d**) KEGG pathway enrichment analysis of the DAMs. The *x*-axis represents the enrichment significance (−Log_10_(adjusted *p*-value)), and the bar colors indicate different metabolic categories.

A total of 1233 metabolites were identified and classified into various chemical subclasses. The most abundant categories included lipids and lipid-like molecules (23.0%), organic acids and derivatives (19.5%), phenylpropanoids and polyketides (16.6%), and organoheterocyclic compounds (14.9%) (Figure S1a). To visualize the overall differences in metabolic profiles among the samples, principal component analysis (PCA) was performed. The PCA score plot revealed that biological replicates within each group clustered tightly together, indicating high reproducibility of the data. The first two principal components, PC1 and PC2, explained 21.6% and 12.2% of the total variance, respectively. Notably, the samples were clearly separated along the PC1 axis based on genotype, with the wild-type W82 samples clustering on the left side and the *sss1* mutant samples on the right. This distinct segregation suggests that the *sss1* mutation significantly disrupts metabolic homeostasis. Additionally, a separation trend along the PC2 axis was observed, particularly distinguishing the 24 h time point (blue and teal) from the 0 h and 3 h time points, reflecting the metabolic shifts in response to salt stress duration (Figure 2a).

To characterize the metabolic reprogramming induced by genotype and salt stress duration, we quantified the number of differentially accumulated metabolites (DAMs) across various comparison groups. As shown in Figure S1b, a substantial number of DAMs were identified in all comparisons. In the comparison between the wild-type W82 and the *sss1* mutant, the number of upregulated metabolites consistently exceeded the downregulated ones. Specifically, 525 metabolites were upregulated and 355 were downregulated at 0 h, which increased to 551 upregulated and 389 downregulated at 3 h and peaked at 581 upregulated and 358 downregulated at 24 h. In the temporal analysis of the wild-type W82 under salt stress, the number of DAMs showed a dynamic change. At 3 h of salt treatment, 394 metabolites were upregulated and 297 were downregulated compared to the 0 h control. By 24 h, the number of upregulated metabolites increased to 464, while the downregulated metabolites rose to 372, indicating a progressive metabolic adjustment to prolonged salt stress. A core set of 431 metabolites was commonly regulated in response to salt stress and genotype differences. However, unique sets of metabolites were specific to certain comparisons; for instance, 30 metabolites were uniquely altered in the W82 vs. *sss1* comparison at 24 h, while 24 were unique to the W82 0 h vs. 24 h comparison (Figure 2b).

Hierarchical clustering analysis (HCA) of the DAMs clearly segregated the samples into distinct groups corresponding to genotype and treatment duration, confirming the reproducibility of the data and the distinct metabolic signatures of W82 and *sss1* under salt stress (Figure 2c). To understand the biological processes involved, KEGG pathway enrichment analysis was performed. The DAMs were significantly enriched in pathways related to metabolism, including biosynthesis of amino acids, isoflavonoid biosynthesis, 2-Oxocarboxylic acid metabolism, and ABC transporters. Additionally, pathways related to environmental information processing and genetic information processing (e.g., Aminoacyl-tRNA biosynthesis) were also significantly enriched, suggesting that *sss1* has altered metabolic flux and stress response capabilities compared to the wild type (Figure 2d).

### 2.3. Transcriptomic Response to Salt Stress

To elucidate the molecular mechanisms underlying the salt sensitivity of the sss1 mutant, we performed a comparative transcriptome analysis of W82 and sss1 seedlings under salt stress at 0 h, 3 h, and 24 h.

**Figure 3:**
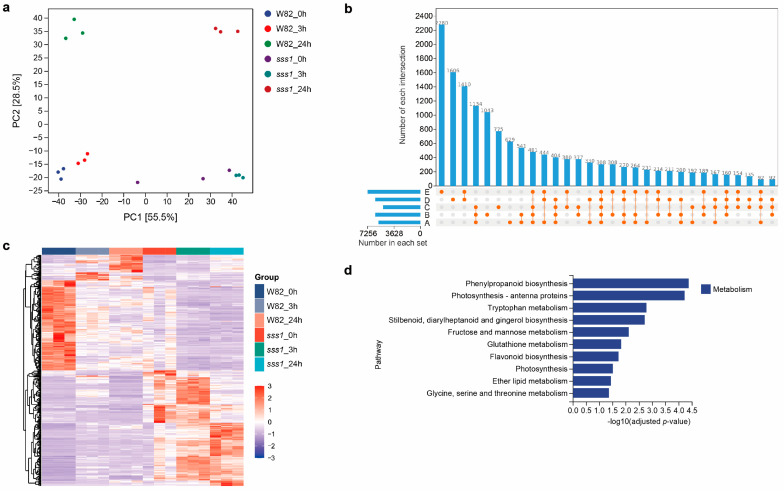
Transcriptomic analysis of differentially expressed genes (DEGs) between W82 and *sss1*. (**a**) Principal component analysis (PCA) plot of all samples based on gene expression levels. PC1 and PC2 explain 55.5% and 28.5% of the total variance, respectively. Samples cluster by genotype and time point. (**b**) UpSet plot showing the overlap of DEGs across five comparison groups. The specific comparisons are: (A) W82 vs. *sss1* at 0 h; (B) W82 vs. *sss1* at 3 h; (C) W82 vs. *sss1* at 24 h. Panels (D) and (E) represent the intra-group comparisons (replicates) for W82 at 3 h and 24 h, respectively. (**c**) Hierarchical clustering heatmap of the expression levels of 308 DEGs. The color scale represents the relative expression level (z-score). (**d**) KEGG pathway enrichment analysis of the DEGs. The *x*-axis represents the enrichment significance (−Log_10_(adjusted *p*-value)).

Principal component analysis (PCA) revealed distinct separation of samples along PC1 (55.5%) and PC2 (28.5%). The biological replicates for each group clustered tightly, while the samples treated with salt (3 h and 24 h) were clearly separated from the untreated controls (0 h) along the PC2 axis. Notably, the sss1 mutant samples exhibited a distinct separation from the W82 samples, indicating significant transcriptional divergence between the genotypes in response to salt stress (Figure 3a). Quantitative analysis of differentially expressed genes (DEGs) revealed extensive transcriptional reprogramming in response to both genotype and salt stress duration. Comparative analysis between the wild-type W82 and the *sss1* mutant under control conditions (0 h) identified 3066 upregulated and 2692 downregulated genes, indicating inherent transcriptional differences between the genotypes. Upon salt treatment, the divergence in gene expression became more pronounced. At 3 h of salt stress, the comparison between W82 and *sss1* yielded 3015 upregulated and 3181 downregulated DEGs. Interestingly, at 24 h, the number of downregulated genes in the mutant comparison decreased to 1963, while upregulated genes remained high at 3148 (Figure S2). In the wild-type W82, the temporal response to salt stress was characterized by a dominance of downregulated genes. Comparing W82 at 3 h versus 0 h resulted in 2417 upregulated and 3780 downregulated genes. This trend was further amplified at 24 h, where the number of downregulated genes peaked at 4351, significantly exceeding the 2905 upregulated genes (Figure S2). These results suggest that while the mutant exhibits a distinct transcriptional profile compared to the wild type, the wild-type response to prolonged salt stress involves a massive suppression of gene expression. UpSet plots highlighted that a core set of 308 genes was commonly regulated in response to salt stress in both genotypes (Figure 3b). Hierarchical clustering of these DEGs confirmed the distinct transcriptional responses between the wild type and the mutant (Figure 3c). KEGG enrichment analysis of the DEGs pointed to phenylpropanoid biosynthesis, photosynthesis antenna proteins, and fructose and mannose metabolism as significantly affected pathways (Figure 3d), providing insights into the molecular mechanisms underlying the observed phenotypic differences.

### 2.4. Insights from Integrated Transcriptome and Metabolome

**Figure 4:**
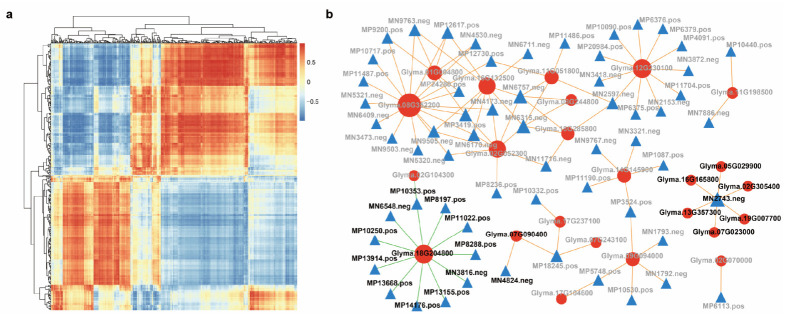
Integrated transcriptomic and metabolomic analysis. (**a**) Hierarchical clustering heatmap illustrating the correlation between 308 differentially expressed genes (DEGs) and 431 differentially accumulated metabolites (DAMs). Pearson correlation coefficients (PCC) were calculated using the R cor function, and pairs with |PCC| > 0.8 were selected. The color scale represents the correlation coefficient, ranging from negative (blue) to positive (red). (**b**) Co-expression network analysis of the top correlated gene-metabolite pairs. The top 100 gene-metabolite pairs with the highest |PCC| values were selected for network visualization. The network consists of nodes and edges, where red circular nodes represent genes, blue triangular nodes represent metabolites, and the connecting lines represent significant correlations. The color of the lines indicates a negative (green) or positive (orange) correlation. The black font represents the regulatory modules of primary interest, while the gray font indicates others.

To elucidate the complex regulatory networks underlying the salt stress response, an integrated analysis of 431 differentially accumulated metabolites (DAMs) and 308 differentially expressed genes (DEGs) was performed. Hierarchical clustering analysis revealed distinct expression and accumulation patterns, grouping the data into several major clusters characterized by contrasting positive-regulation (red) and negative-regulation (blue) trends (Figure 4a). This segregation indicates a coordinated transcriptional and metabolic reprogramming between W82 and *sss1* in response to salinity.

Furthermore, a correlation network was constructed to visualize specific gene-metabolite interactions in the co-expression network analysis (Figure 4b). In this network, nodes represent genes (circles) and metabolites (triangles), while the connecting lines indicate significant correlations. The analysis highlighted several regulatory modules, most notably a distinct cluster centered around mannose (MN2743.neg). This metabolite acts as a key metabolic node, showing strong positive correlations with genes involved in carbohydrate metabolism and glycosylation (such as *Glyma.02G305400*, *Glyma.07G023000*, *Glyma.13G357300*, *Glyma.16G165800*, and *Glyma.19G007700*) (Figure 4b), suggesting that mannose-mediated signaling or cell wall modification plays a pivotal role in the early response to salt stress.

Additionally, a highly interconnected module was identified involving FA 18:1 (oleic acid, MN6548.neg) and 12-Oxo phytodienoic acid (OPDA, MP10250.pos). These metabolites were negatively correlated with the gene *Glyma.18G204800* (Figure 4b). Given that OPDA serves as a precursor to jasmonates, this network highlights the coordinated changes in fatty acid metabolism and the jasmonic acid pathway underlying the salt stress response of the *sss1* mutant.

Moreover, a specific sub-network connected Abscisic acid (ABA, MN4824.neg) with the gene *Glyma.07G090400* (Figure 4b). This interaction underscores the involvement of ABA-dependent signaling pathways, which are crucial for stomatal regulation and osmotic adjustment under saline conditions.

Overall, these integrated results suggest that the differential salt tolerance involves a complex interplay between hormone signaling (ABA, Jasmonates), energy metabolism (Mannose), and membrane lipid remodeling.

### 2.5. Comparative Profiling of Specific Metabolites and Genes

To further investigate the metabolic alterations and transcriptional regulation suggested by the integrated network analysis, we extracted and analyzed the relative abundance profiles of five specific metabolites and the expression patterns of representative genes in W82 and the *sss1* mutant under salt stress.

**Figure 5:**
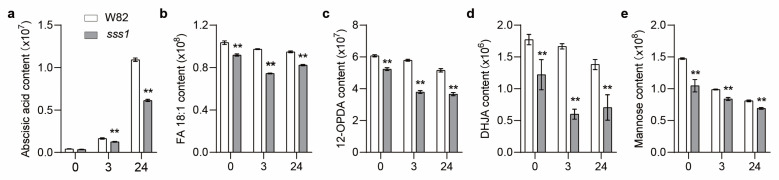
Comparative profiling of specific metabolites in W82 and *sss1* under salt stress. The relative content (measured as the relative ion signal intensity) of Abscisic acid (ABA) (**a**), FA 18:1 (**b**), 12-OPDA (**c**), DHJA (**d**), and mannose (**e**) was measured in W82 (black bars) and sss1 (gray bars) plants at 0, 3, and 24 h after NaCl treatment. Data are presented as means ± SD (*n* = 5). Asterisks indicate statistically significant differences between W82 and *sss1* under the same condition (** *p* < 0.01, Student’s *t*-test).

Quantitative analysis showed that salt treatment triggered a significant increase in Abscisic acid (ABA) content (relative ion signal intensity) in both genotypes. However, the accumulation of ABA was markedly higher in W82 compared to the *sss1* mutant, particularly at the 24 h time point, where W82 exhibited a dramatic surge that was attenuated in the mutant. Similarly, for FA 18:1 (Figure 5b) and 12-OPDA (Figure 5c), W82 generally maintained higher levels or showed a more robust response compared to *sss1*, although the differences were less pronounced than those observed for ABA. In contrast, the levels of DHJA (Figure 5d) and mannose (Figure 5e) displayed a decreasing trend over time in both genotypes under salt stress. Notably, the *sss1* mutant consistently exhibited significantly lower levels of both DHJA and mannose compared to W82 across all time points (0, 3, and 24 h). These results reveal distinct metabolic and transcriptional profiles associated with the *sss1* mutation under salt stress. We observed a correlation between the *sss1* genotype and altered accumulation patterns of ABA, JA metabolites, and mannose.

Genes involved in ABA biosynthesis (e.g., *NCED1*, *NCED3*, *NCED4*) and ABA signaling (e.g., *ABF1*, *ABF2*, *PYL8*, *PYL9*) exhibited distinct expression dynamics. Notably, *NCED1* (*Glyma.13G202200*) showed a pronounced upregulation in W82 at 24 h (377.1), contrasting with lower expression in *sss1* (159.7). Conversely, the expression of *NCED4* (*Glyma.01G154900*) was higher in the wild-type W82 (348.9) than in the *sss1* mutant (260.9) at the basal level (0 h). Under salt stress, *NCED4* expression declined in both genotypes, but the mutant exhibited a sharper decrease over time. Although the two *NCED3* homologs (*Glyma.08G176300* and *Glyma.15G250100*) showed low overall expression in both genotypes, W82 exhibited a salt-induced upregulation trend with higher expression levels compared to the mutant. Consistent with the elevated ABA levels, the ABA-responsive genes *COR47* (*Glyma.04G009400*) and *Dehydrin* (*Glyma.07G090400*) were more strongly induced in W82 than in *sss1*, indicating a robust ABA signaling response in the wild type that is compromised in the mutant.

**Figure 6:**
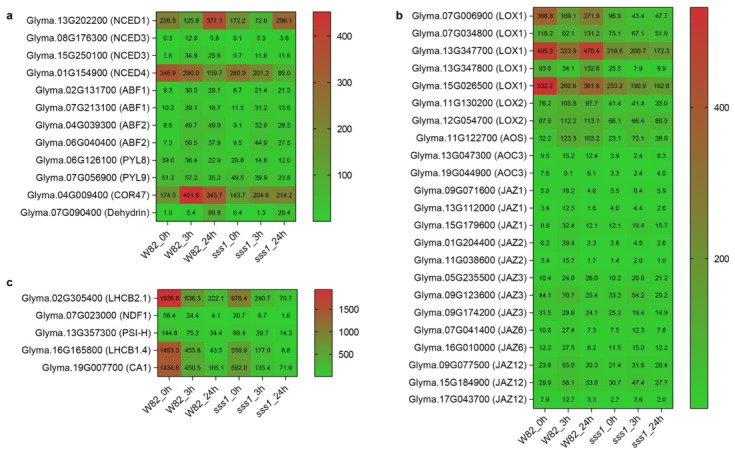
Expression profiles of genes involved in hormone signaling and photosynthesis. Heatmaps display the expression levels (FPKM values) of selected differentially expressed genes (DEGs) in W82 and *sss1* samples at 0, 3, and 24 h after NaCl treatment: (**a**) Expression profiles of genes involved in the ABA biosynthesis and signaling pathway, including *NCEDs*, *ABFs*, *PYLs*, *COR47*, and *Dehydrin*. (**b**) Expression profiles of genes involved in the JA biosynthesis and signaling pathway, specifically *LOX*, *AOS*, *AOC*, and *JAZ* gene families. (**c**) Expression profiles of genes related to photosynthesis, including *LHCBs*, *NDF*, *PSI-H*, and *CA1*. The color scale represents the expression level, with red indicating high expression and green indicating low expression. The specific FPKM values are indicated within each cell, and these values represent the mean of three biological replicates.

The expression of genes involved in JA biosynthesis and signaling showed a clear genotype-dependent response (Figure 6b). JA biosynthesis genes, including multiple *LOX* (e.g., *Glyma.07G006900*, *Glyma.07G034800*, *Glyma.11G130200*, *Glyma.11G130200*, *Glyma.12G054700*, *Glyma.13G347700*, *Glyma.13G347800*, *Glyma.15G026500*), *AOS* (*Glyma.11G122700*), and *AOC* (*Glyma.13G047300*, *Glyma.19G044900*) homologs, showed much higher expression levels in W82 throughout the treatment. Similarly, genes encoding JAZ repressors (*JAZ1*, *JAZ2*, *JAZ3*, *JAZ6*, *JAZ12*) generally exhibited higher expression in W82. These results suggest a defect in the JA biosynthesis and signaling pathway in the *sss1* mutant compared to the wild type.

Consistent with the genes described above, five genes associated with the photosynthetic machinery, specifically *LHCB2.1* (*Glyma.02G305400*), *NDF1* (*Glyma.07G023000*), *PSI-H* (*Glyma.13G357300*), *LHCB1.4* (*Glyma.16G165800*), and *CA1* (*Glyma.19G007700*), exhibited significantly higher expression in W82 compared to the *sss1* mutant, with expression levels differing by more than two-fold (Figure 6c). During the salt stress treatment, the transcript abundance of these genes showed a time-dependent decrease in both genotypes (Figure 6c). These findings suggest that both the *sss1* mutation and salt stress contribute to the transcriptional suppression of these photosynthesis-related genes.

Relative expression levels of six representative genes were selected and analyzed by quantitative real-time PCR (RT-qPCR) to validate the reliability of the transcriptomic data (Figure S3). The results confirm the expression trends observed in the RNA-seq analysis, supporting the accuracy of the transcriptomic profiling.

## 3. Discussion

Salt stress is a major environmental constraint limiting soybean productivity, and understanding the genetic and molecular basis of salt tolerance is essential for crop improvement. In this study, we combined physiological, metabolomic, and transcriptomic analyses to dissect the mechanisms underlying salt sensitivity in the *sss1* mutant. Our findings reveal that the reduced tolerance of *sss1* is not attributable to a single defect but instead arises from coordinated disruptions in ion homeostasis, metabolic reprogramming, hormone signaling, and energy allocation.

### 3.1. Disrupted Ion Homeostasis as the Primary Cause of Salt Sensitivity in sss1

The maintenance of intracellular ionic homeostasis, particularly a low cytosolic Na^+^/K^+^ ratio, is a hallmark of salt-tolerant glycophytes [38,39]. The *sss1* mutant exhibited a characteristic ion-toxic phenotype, characterized by excessive Na^+^ accumulation and impaired K^+^ retention, resulting in a markedly elevated Na^+^/K^+^ ratio. Such ionic imbalance is cytotoxic, as it disrupts enzyme activity, protein synthesis, and membrane integrity, ultimately leading to the observed growth inhibition and chlorosis [1].

Mechanistically, Na^+^ homeostasis is maintained through the coordinated activity of transport systems, including the plasma membrane Na^+^/H^+^ antiporter (SOS1), vacuolar NHX exchangers, and HKT-mediated Na^+^ retrieval from the xylem stream [5,12,13]. The severe ion imbalance in *sss1* suggests that the *SSS1* locus is intimately involved in the regulation of ion transport systems. The *sss1* mutation is associated with a failure to maintain Na^+^/K^+^ homeostasis, which likely acts as a primary upstream signal triggering the downstream metabolic and transcriptional changes observed in this mutant [9]. Thus, the disruption of ion homeostasis likely represents the initiating event in the cascade of dysfunctions observed in the *sss1* mutant.

Salt stress involves both osmotic and ionic components. In this study, we focused primarily on ion homeostasis and specific signaling mechanisms. Therefore, we did not measure water potential or relative water content (RWC). However, the severe growth inhibition and morphological changes observed in *sss1* (Figure 1) clearly indicate a significant stress burden. These phenotypes serve as indirect evidence that the plants suffered from substantial physiological constraints. Furthermore, our transcriptomic data revealed the upregulation of several stress-responsive genes (e.g., *COR47* and *Dehydrin*), which are often associated with osmotic adjustment pathways, suggesting that the observed transcriptional changes capture the plant’s integrated response to both ionic and osmotic stress. We acknowledge this limitation and suggest that future studies should include direct measurements of water status to fully distinguish the osmotic and ionic phases of the stress response.

### 3.2. Metabolic Reprogramming and the Emerging Role of Mannose

Metabolic adjustment is a critical survival strategy, enabling plants to achieve osmotic balance and detoxify reactive oxygen species (ROS) [25,40]. While sucrose and trehalose are well-established regulators of stress signaling [41,42], the prominence of mannose suggests a potential regulatory role in soybean salt responses.

Mannose is a critical substrate for N-glycosylation, a post-translational modification essential for the proper folding, stability, and function of glycoproteins [43]. Our integrated analysis identified mannose metabolism as a special node that is significantly altered in the *sss1* mutant. The strong correlation between reduced mannose levels and salt sensitivity suggests that mannose metabolism may represent a metabolic vulnerability in this genotype. While the exact role of mannose requires functional validation, its association with protein glycosylation pathways implies a potential mechanism underlying the observed stress sensitivity.

### 3.3. Altered Hormone Accumulation and Gene Expression Profiles in sss1

Phytohormones serve as master integrators of environmental cues [30,44]. Abscisic acid (ABA) is a key regulator of osmotic stress responses, controlling stomatal closure and the induction of protective genes [26,45]. JA plays crucial roles in membrane stabilization and ROS regulation [28,29,46]. Importantly, ABA and JA pathways are interconnected; they often act synergistically to coordinate stress responses [30,31].

The transcriptomic and metabolomic data revealed significant differences in the ABA and JA pathways between W82 and *sss1*. The *sss1* mutant exhibited a distinct metabolic profile characterized by a reduced capacity to accumulate ABA and DHJA under stress. Concomitantly, the expression of biosynthetic genes (*NCEDs*, *LOXs*, and *AOS*) was significantly lower in the mutant compared to the wild type.

These correlative data suggest that the *sss1* mutation may affect the hormonal response machinery. However, we acknowledge that without exogenous hormone application (rescue) experiments, we cannot definitively conclude that the hormonal pathways are the primary cause of the salt-sensitive phenotype. Future functional studies will be necessary to determine whether restoring hormone levels can rescue the *sss1* phenotype.

### 3.4. Photosynthetic Adjustment and Energy Allocation Constraints

The downregulation of photosynthesis is a common adaptive strategy to minimize ROS production under stress [47,48]. In the wild type, the suppression of light-harvesting complex (*LHCB*) genes reflects a controlled reduction in energy input. The *sss1* mutant, however, exhibited dysregulated expression of these genes, indicating a failure in photosynthetic responses. Furthermore, the differential expression of carbonic anhydrase (*CA1*) suggests perturbations in CO_2_ assimilation and pH regulation.

This reflects a broader failure in energy reallocation. Efficient stress tolerance requires the prioritization of maintenance and defense over growth [49]. The inability of *sss1* to achieve this metabolic shift—optimizing energy use from growth to stress defense—likely contributes significantly to its reduced survival under saline conditions.

## 4. Materials and Methods

### 4.1. Plant Materials and Growth Conditions

The soybean (*Glycine max* L. Merr.) salt-sensitive mutant *sss1* was isolated from an ethyl methanesulfonate (EMS)-mutagenized M_2_ population derived from the cultivar Williams 82 (W82). Sterilized soybean seeds were germinated and grown in moist vermiculite until the cotyledons expanded at 25 °C. Uniform seedlings were selected and hydroponically cultivated in a controlled-environment growth chamber with a 16 h light (about 350 μmol m^−2^ s^−1^)/8 h dark cycle at 25 °C. Plants were grown in 1/2-strength Hoagland’s nutrient solution, which was renewed every three days.

### 4.2. Salt Stress Treatment and Phenotypic Evaluation

W82 seedlings at the V1 growth stage (fully expanded first trifoliate leaf) were treated with 1/2-strength Hoagland solution supplemented with 150 mM NaCl (Sinopharm, Shanghai, China). For omics analyses, fully expanded first trifoliate leaf samples were harvested at 0, 3, and 24 h after treatment. Five biological replicates (two seedlings pooled per replicate) were collected for metabolomics, and three biological replicates were harvested for RNA-seq. All samples were harvested from the same pot and flash-frozen in liquid nitrogen and stored at −80 °C.

For phenotypic and physiological assays, W82 and *sss1* seedlings grown in the same pot were treated for 5 days alongside NaCl-free controls. Following treatment, leaf, stem, and root tissues were collected. Traits including fresh weight (FW), dry weight (DW), and chlorophyll content were recorded. Chlorophyll content was measured using a SPAD-502 Plus chlorophyll meter (Konica Minolta, Osaka, Japan). Additionally, approximately 0.1 g of finely ground dry tissue was digested in 5 mL of 65% HNO_3 65%_ (analytical grade, Sinopharm, Shanghai, China) at 90 °C for 6–8 h and diluted to 25 mL to quantify Na^+^ and K^+^ concentrations using ICP-OES (PerkinElmer, Shelton, CT, USA) [50], from which the Na^+^/K^+^ ratio was calculated.

### 4.3. Metabolomics Analysis

Frozen tissues were sent to Personalbio (Shanghai) Co., Ltd. (Shanghai, China) for metabolite detection. Samples ground in liquid nitrogen (0.1 g for each) were extracted using a pre-cooled methanol–acetonitrile–water solution (2:2:1, Fisher Scientific, Waltham, MA, USA). Mass spectrometry data were acquired in both positive and negative electrospray ionization (ESI) modes using a Thermo Orbitrap Exploris 120 (Thermo Fisher Scientific, Waltham, MA, USA) controlled by Xcalibur software (v4.7, Thermo Fisher Scientific), operating in data-dependent acquisition (DDA) mode. Instrument stability was monitored throughout the run using quality control (QC) samples.

Raw data were processed in MS-DIAL software (version 4.9.221218, RIKEN Center for Sustainable Resource Science, Yokohama, Japan) for peak extraction and normalization. Metabolites were identified against the PSNGM database (Personalbio, Shanghai, China), which includes self-built libraries, mzCloud, LIPIDMAPS, HMDB, MoNA, NIST_2020, and AI-predicted MS/MS spectral libraries.

Data analysis was conducted based on the Personalbio Genescloud platform (http://www.genescloud.cn, accessed on 13 October 2025). The data matrix was imported into R (version 4.3.3) for principal component analysis (PCA) to assess the overall distribution of samples and the stability of the analytical process. Subsequently, orthogonal partial least squares discriminant analysis (OPLS-DA) and partial least squares discriminant analysis (PLS-DA) were employed to identify differentially accumulated metabolites between groups.

To ensure model reliability and prevent overfitting, we performed 7-fold cross-validation and 200 response permutation tests (RPTs). The contribution of variables to group separation was ranked using variable importance in the projection (VIP) values obtained from the OPLS-DA model. Combined with a two-tailed Student’s *t*-test, metabolites with VIP > 1.0 and adjusted *p*-value < 0.05 were classified as differentially accumulated metabolites (DAMs). These DAMs were subsequently visualized via Upset diagrams and hierarchical clustering heatmaps and functionally annotated through KEGG pathway enrichment analysis (clusterProfiler, V4.6.0).

### 4.4. RNA Extraction, Library Construction, Transcriptome Sequencing, and RT-qPCR

Total RNA was extracted from approximately 0.1 g of tissue powder ground in liquid nitrogen using TRIzol™ Reagent (Invitrogen Life Technologies, Carlsbad, CA, USA) according to the manufacturer’s instructions. RNA concentration, purity, and integrity were assessed using a NanoDrop spectrophotometer (Thermo Fisher Scientific, Wilmington, DE, USA).

For library preparation, a total of 1 µg of high-quality RNA per sample was used as input material. Libraries were constructed using the NEBNext Ultra II RNA Library Prep Kit (New England Biolabs Inc., Ipswich, MA, USA). The libraries were sequenced on an Illumina NovaSeq 6000 platform (Illumina, San Diego, CA, USA) to generate 150-bp paired-end reads.

Raw sequencing data were processed using FastP (v0.22.0) to filter out low-quality reads and adapters, producing clean reads for downstream analysis. Clean reads were aligned to the *Glycine max* reference genome (Wm82.a4.v1) using HISAT2 (v2.1.0). Read counts were quantified using HTSeq (v0.9.1). Expression levels were normalized as Fragments Per Kilobase of transcript per Million mapped reads (FPKM). Differential gene expression analysis was performed using DESeq2 (v1.38.3) with raw count data as input. Differentially expressed genes (DEGs) were identified with thresholds of |log2FoldChange| > 1 and adjusted *p*-value < 0.05. To visualize the overlap of DEGs across different comparisons, UpSet plots were generated using the UpSetR package (v1.4.0). Bidirectional clustering analysis was subsequently performed on these DEGs using the ComplexHeatmap package (v2.16.0). The heatmap was generated based on Z-score-normalized FPKM values, with Euclidean distance and complete linkage methods applied for distance calculation and clustering, respectively. Functional enrichment analysis of differentially expressed genes was carried out using ClusterProfiler (v4.6.0) to identify significantly enriched KEGG pathways (adjusted *p*-value < 0.05).

For RT-qPCR analysis, cDNA was synthesized from the RNA using HiScript II Q Select RT SuperMix (Vazyme, Nanjing, China), following the manufacturer’s instructions. RT-qPCR was performed on a QuantStudio™ 5 Real-Time PCR System (Applied Biosystems, Chicago, IL, USA) using the ChamQ Universal SYBR qPCR Master Mix (Vazyme, Nanjing, China). The relative expression of target genes was calculated using the 2^−ΔΔCT^ method, with GmActin11 transcript levels as the internal control. Data were presented as the mean of three biological replicates. Gene-specific primers are listed in Supplementary Table S7.

### 4.5. Strategy for Integrated Transcriptome and Metabolome Analysis

To elucidate the interactions between transcriptional and metabolic profiles, we performed a correlation analysis using the 431 differentially accumulated metabolites and 308 differentially expressed transcripts derived from multi-omics screening. For each comparative group, Pearson correlation coefficients (PCCs) were calculated between the filtered metabolites and genes using the cor function in R (version 4.3.3), specifically selecting gene-metabolite pairs with |PCC| > 0.8 to ensure strong associations. The differentially expressed genes (DEGs) were identified with a significance threshold of |log2FoldChange| > 1 and adjusted *p*-value < 0.05, while the differentially accumulated metabolites (DAMs) were screened using criteria of variable importance in projection (VIP) > 1 and adjusted *p*-value < 0.05.

To visualize the coordinated patterns, a clustering heatmap was generated based on the correlation coefficients. Furthermore, to construct a correlation network highlighting the most significant interactions, the top 100 gene-metabolite pairs with the highest |PCC| values were selected for network visualization.

## 5. Conclusions and Future Perspectives

In this study, we characterized the EMS-derived soybean salt-sensitive mutant *sss1*. Integrated analysis suggests that the salt sensitivity of *sss1* involves dysregulated Na^+^/K^+^ homeostasis, altered mannose metabolism, and perturbations in ABA/JA signaling pathways. These observations identify candidate mechanisms associated with the *sss1* phenotype and establish a basis for the future cloning of *SSS1* and detailed functional validation.

Given that *sss1* was isolated from an EMS-mutagenized population, the potential influence of background mutations cannot be ruled out. Therefore, while our data establish a strong correlation between the *sss1* genotype and the observed stress defects, the current evidence describes the mutant’s phenotypic features rather than definitively proving the exclusive molecular function of the *SSS1* gene.

Future research will focus on bridging this gap between phenotype and genotype. Priorities include identifying the causal mutation through MutMap and validating the gene’s function via genetic complementation or CRISPR/Cas9-mediated knockout. Confirming *SSS1* as a key regulator of salt tolerance will not only clarify its molecular mechanism but also provide valuable genetic resources for breeding salt-resistant soybean varieties.

## Data Availability

All data generated and analyzed are included in this manuscript and its Supplementary Materials.

## Supplementary Materials

The following supporting information can be downloaded at: https://www.mdpi.com/article/10.3390/plants15111695/s1. Figure S1. Overview of metabolite identification and variation. (a) Pie chart showing the percentage distribution of 1233 identified metabolites across different chemical classes. (b) Number of significantly up-regulated (red) and down-regulated (blue) metabolites in pairwise group comparisons; Figure S2. Number of up-regulated (red) and down-regulated (blue) DEGs in pairwise comparisons between different groups; Figure S3. RT-qPCR validation of selected differentially expressed genes (DEGs). Relative expression levels of six candidate genes (*Glyma.15G250100*, *Glyma.04G009400*, *Glyma.07G006900*, *Glyma.15G026500*, *Glyma.02G305400*, and *Glyma.19G007700*) in Figure 6 were analyzed by RT-qPCR in wild-type W82 (white bars) and *sss1* mutant (gray bars) roots at 0, 3, and 24 h after 150 mM NaCl treatment. Expression levels were normalized to the Actin reference gene and are shown as mean ± SD of three biological replicates; Supplemental Table S1. Metabolomic Profiling of W82 and *sss1*; Supplemental Table S2. Transcriptome data of W82 and *sss1*; Supplemental Table S3. A core set of 431 metabolites were commonly regulated in response to salt stress in W82 and *sss1*; Supplemental Table S4. A core set of 308 genes were commonly regulated in response to salt stress in W82 and *sss1*; Supplemental Table S5. Correlation analysis of differentially expressed genes and metabolites with correlation coefficients > 0.8; Supplemental Table S6. Top 100 correlation network based on the lowest *p*-values; Supplemental Table S7. Primers used in this study.
