# Supplementary material for: Integrated Multi-Omics Characterization of the Salt-Sensitive Mutant sss1 in Soybean
Source: Plants (Basel). 2026 May 30;15(11):1695. doi: 10.3390/plants15111695 (PMC13258952; doi:10.3390/plants15111695)
Supplement: Supplementary file 1 [file plants-15-01695-s001.zip › Figure S1-3.pptx]

## Slide 1
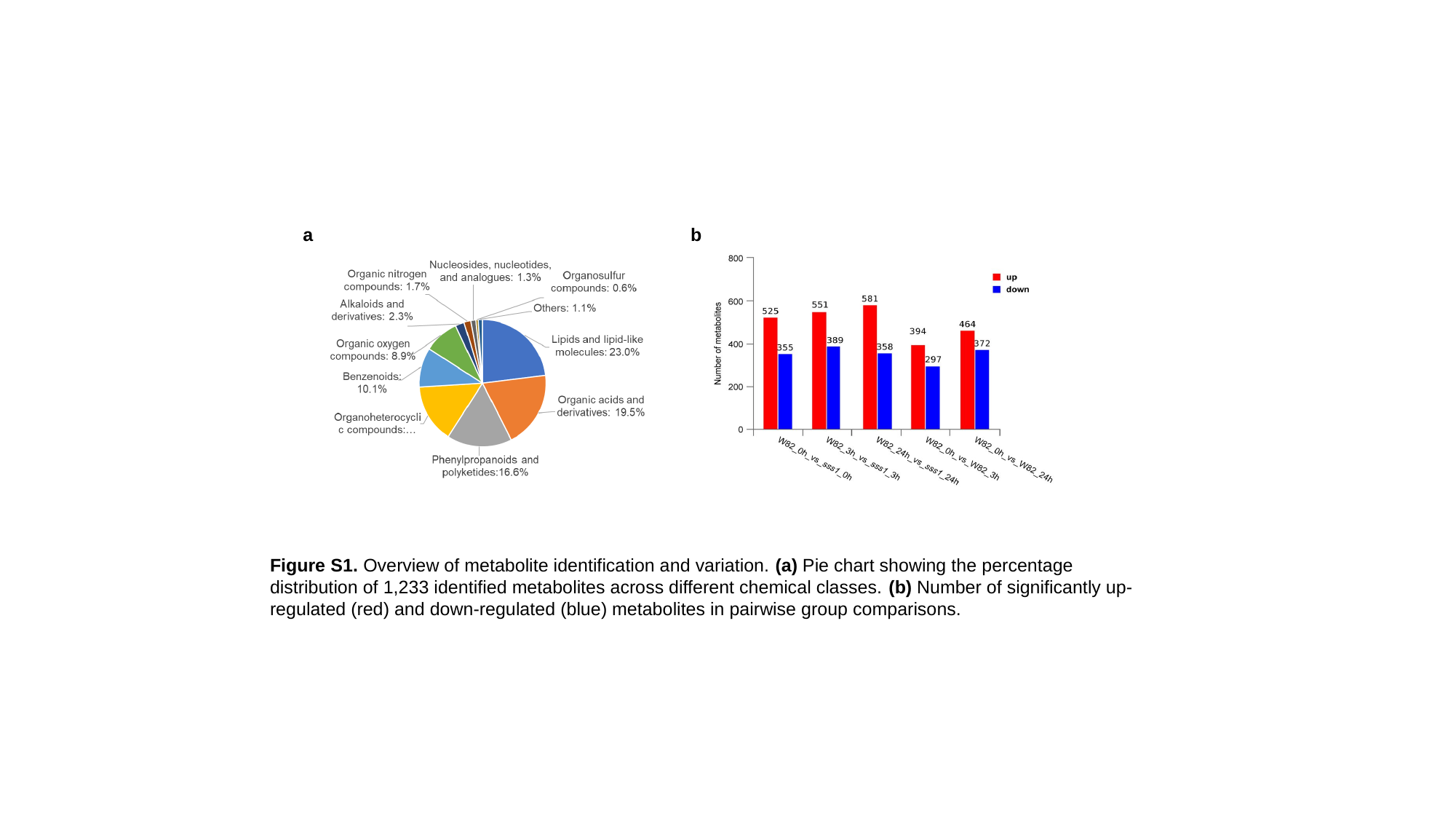

a
b
Figure S1. Overview of metabolite identification and variation. (a) Pie chart showing the percentage distribution of 1,233 identified metabolites across different chemical classes. (b) Number of significantly up-regulated (red) and down-regulated (blue) metabolites in pairwise group comparisons.

## Slide 2
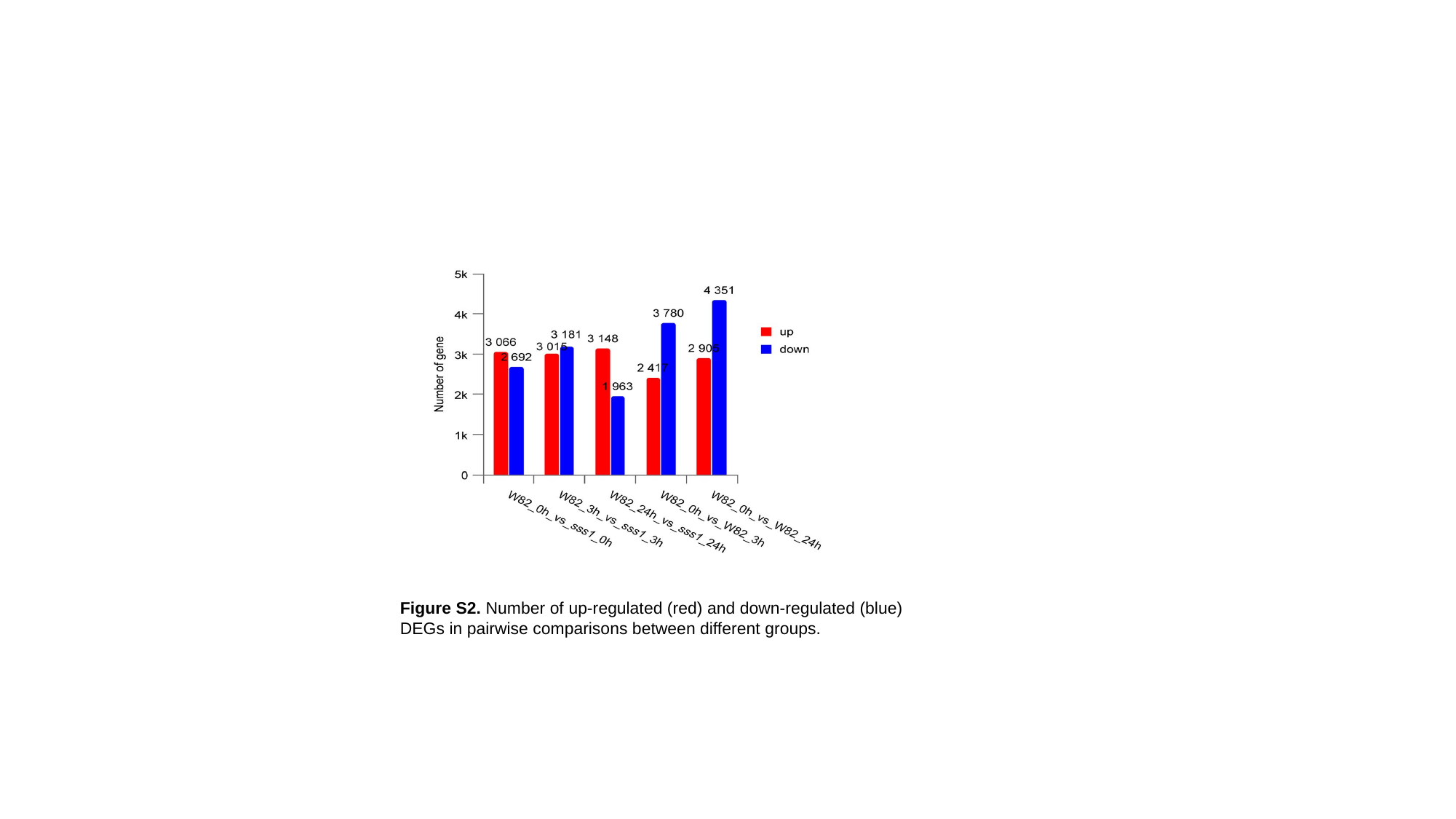

Figure S2. Number of up-regulated (red) and down-regulated (blue) DEGs in pairwise comparisons between different groups.

## Slide 3
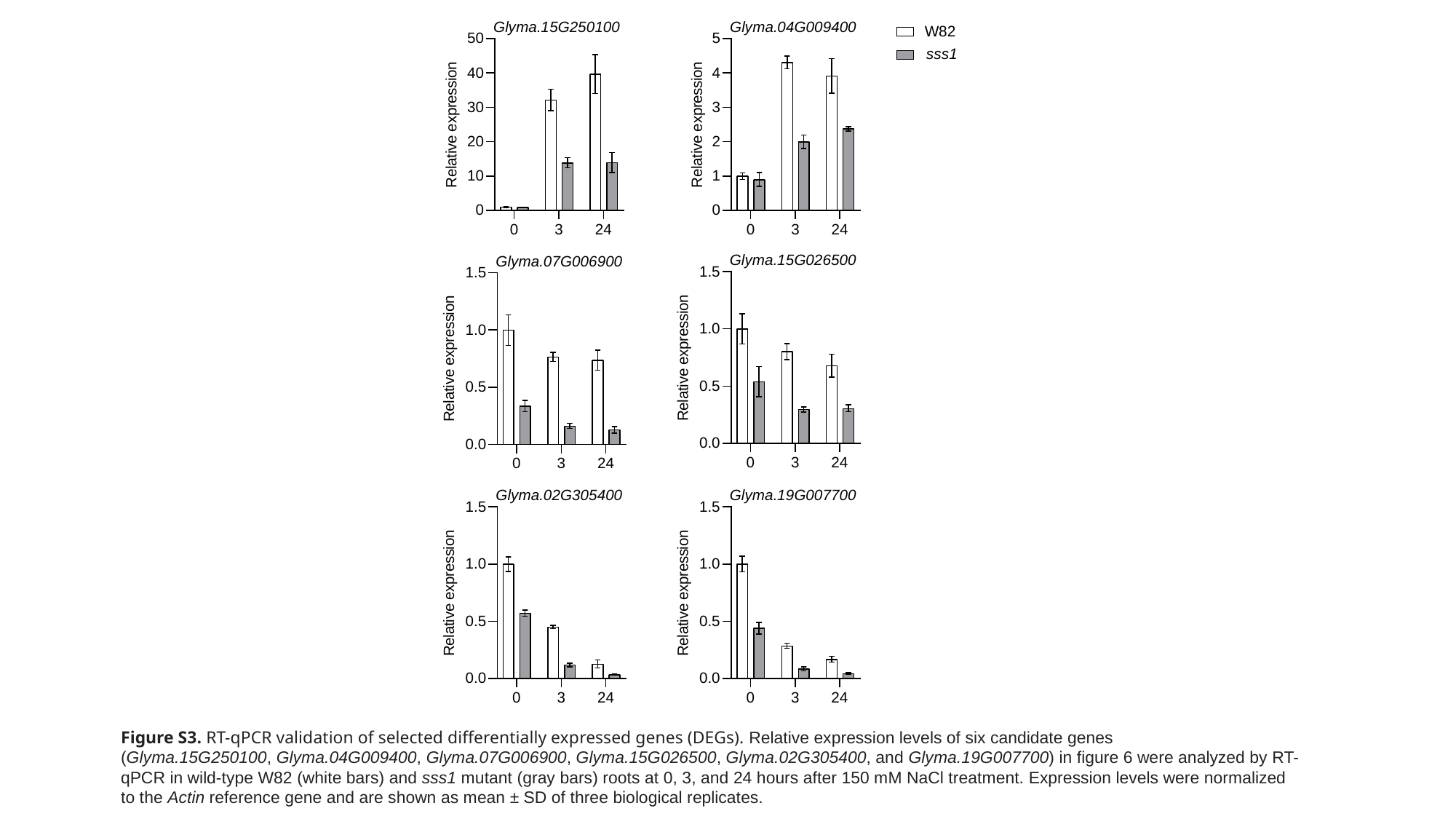

W82
sss1
Figure S3. RT-qPCR validation of selected differentially expressed genes (DEGs). Relative expression levels of six candidate genes (Glyma.15G250100, Glyma.04G009400, Glyma.07G006900, Glyma.15G026500, Glyma.02G305400, and Glyma.19G007700) in figure 6 were analyzed by RT-qPCR in wild-type W82 (white bars) and sss1 mutant (gray bars) roots at 0, 3, and 24 hours after 150 mM NaCl treatment. Expression levels were normalized to the Actin reference gene and are shown as mean ± SD of three biological replicates.
